# Anesthesia management for cesarean section in a woman with chronic renal failure and heart failure: a case report

**DOI:** 10.1186/s13256-024-04694-x

**Published:** 2024-08-05

**Authors:** Tatsuo Horiuchi, Syunsuke Takeda, Rie Mieda, Tadanao Hiroki, Shigeru Saito

**Affiliations:** 1 Department of Anesthesiology, Gunma University Hospital, 3-39-15 Showa-Machi, Maebashi, Gunma 371-8511 Japan; 2 Department of Anesthesiology, Isesaki Municipal Hospital, 12-1 Tsunatorihonmachi, Isesaki, Gunma 372-0817 Japan

**Keywords:** Pregnancy with renal failure, Pregnancy with heart failure, Cesarean section, Combined spinal and epidural anesthesia, Case report

## Abstract

**Introduction:**

Pregnancy in a woman with heart and chronic renal failure can lead to life-threatening complications for both mother and child. Although such cases are often delivered by cesarean section, few reports have described anesthesia methods.

**Case presentation:**

We encountered a case in which cesarean section was performed using combined spinal and epidural anesthesia for a pregnant woman with chronic renal and heart failure. The 35-year-old Japanese woman had been undergoing hemodialysis for several years. Heart failure symptoms that appeared during pregnancy initially improved with treatments such as increasing hemodialysis, but recurred. She was admitted to the intensive care unit. The initial plan was to deliver the baby after a few weeks, but further progression of heart failure became a concern. After a clinical conference among staff, a cesarean section with combined spinal and epidural anesthesia was scheduled for 24 weeks, 0 days of gestation. The anticoagulant for dialysis was also changed from heparin to nafamostat in preparation for cesarean section. Monitoring was started with central venous and radial artery pressures before induction of anesthesia. Combined spinal and epidural anesthesia was induced and the cesarean section was completed without complications. Surgery was initiated under continuous administration of phenylephrine, which was intended to avoid hypotension due to anesthesia. The hemodynamic and respiratory status of the patient remained stable postoperatively. After the cesarean section, morphine was administered epidurally and the epidural catheter was removed.

**Conclusion:**

Cesarean section was safely performed for a pregnant woman with renal and heart failure using combined spinal and epidural anesthesia.

## Background

Pregnancy in a woman with chronic renal failure is considered high risk for both mother and child. Pregnancy complicated by heart failure is likewise regarded as involving increased risks for both mother and child. Pregnant women with renal failure often deliver prematurely, and a cesarean section may be needed. In such cases, problems are seen not only in the method of anesthesia for cesarean section but also in the perioperative management. Anesthesia for cesarean section in pregnancies complicated by renal failure and for cesarean section in pregnancies complicated by heart failure (including perinatal cardiomyopathy) has been reported. However, to the best of our knowledge, there have not been any reported cases of cesarean section in pregnant women with both cardiac and renal failure. We report a case of cesarean section in which the patient with chronic renal failure developed heart failure during pregnancy.

## Case report

Written informed consent for the publication of this report was obtained from the patient. A 35-year-old, Japanese, 42.7-kg (dry weight, 41.1 kg before pregnancy), 147-cm-tall woman was scheduled for cesarean section. She had renal failure due to chronic glomerulonephritis. Before she became pregnant, she had been undergoing hemodialysis three times per week. The hemodialysis access (arteriovenous fistula) was in her left upper arm. The symptoms and course of treatment up to cesarean section are described in Table 1. When the pregnancy was identified, she received hemodialysis five times per week. Although she had not shown any symptoms before the pregnancy, she reported dyspnea at 12 weeks of gestation and subsequently developed severe cough. Transthoracic echocardiography (TTE) showed a cardiac left ventricular ejection fraction (LVEF) of 40% and mild pulmonary hypertension. After hospitalization, hemodialysis was increased to six times per week to prevent heart failure. LVEF improved to almost 50% and symptoms also improved, so the plan was to postpone delivery as long as possible. However, she again developed cough and dyspnea at 22 weeks of gestation.

**Table 1:** Events and symptoms during the pregnancy

Gestational week	Symptoms and events	Test results and treatment
5	Pregnancy	Frequency of dialysis was increased from 3 to 5 times per week
14	Cough and heart failure appeared	TTE showed an EF of 40%. The patient was hospitalized and dialysis frequency was increased to 6 times per week
16	Discharged	TTE showed that LVEF had improved to 50%
22	Heart and respiratory failure appeared	TEE showed an EF of 20% and chest X-ray showed pulmonary congestion. The patient was readmitted to the ICU and she received oxygen at 1 l/minute via nasal cannula. ECUM was performed
24	Cesarean section	

*TTE* transthoracic echocardiography, *LVEF* left ventricular ejection fraction, *ECUM* extracorporeal ultrafiltration method

**Fig. 1:**
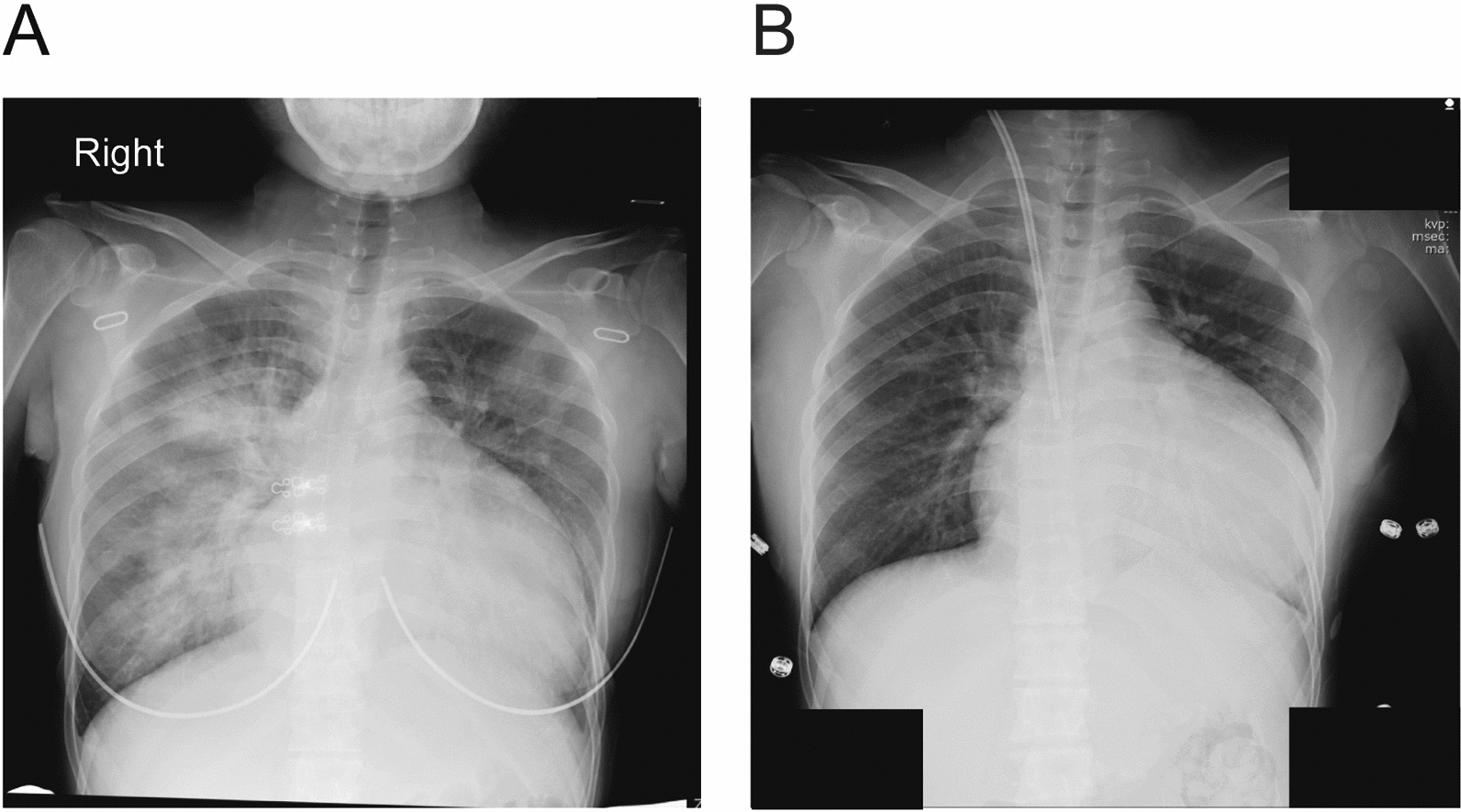
X-ray photographs before and after cesarean section. **A** X-ray at 22 weeks of gestation shows cardiac enlargement and decreased lung field permeability. **B** X-ray immediately after cesarean section shows no change in cardiac enlargement but improved lung field permeability

Chest X-ray showed significant congestion (Fig. 1). TTE showed an LVEF of 20%, mild to moderate mitral regurgitation, and left ventricular dilatation. She was therefore admitted to the intensive care unit (ICU). Percutaneous oxygen saturation (SpO_2_) was 92% on room air, so supplemental oxygen was provided at 1 l/minute through nasal cannula. After oxygen administration, SpO_2_ was 98%. In addition, water volume was removed using extracorporeal ultrafiltration. Although respiratory status and cardiac function improved slightly with intensive care, a clinical conference of obstetricians, pediatricians, urologists, cardiologists, and anesthesiologists decided that cesarean section should be performed at 24 weeks 0 days of gestation. The anticoagulant used for dialysis was changed from heparin to nafamostat mesylate (nafamostat) at 23 weeks of gestation for the cesarean section. Blood tests showed hemoglobin 10.1 g/dl and platelets 12.1 × 10^4^/μl. The prothrombin time international normalized ratio was 0.85, and the activated partial thromboplastin time was 25.6 seconds.

When the patient arrived in the operation room, two intravenous peripheral vein lines were inserted with a 22-gauge cannula and an 18-gauge cannula in the right forearm to use for fluid resuscitation. An arterial line was inserted via the right radial artery with a 22-gauge cannula. Central venous lines were inserted via the jugular vein. We inserted the central venous lines, using the echography while carefully monitoring the arterial line blood pressure. The echography showed the jugular vein clear, and we made sure that putting her in the mild Trendelenburg position did not affect circulatory dynamics. Overall, the procedure was completed without any significant changes in hemodynamics. Spinal and epidural anesthetic agents were administered in the right lateral position at the L3–L4 and Th10–Th11 interspaces, respectively. After local infiltration of 1% mepivacaine, an epidural catheter was placed with an 18-gauge Tuohy needle. The epidural space was confirmed using the conventional loss-off-resistance method. After aspiration testing confirmed a negative result, a test dose of 1% mepivacaine (3 ml) was administered via the catheter. Lumbar puncture was successfully performed, and 5 mg of 0.5% hyperbaric bupivacaine and 10 μg of fentanyl were administered intrathecally with a 25-gauge Quincke spinal needle. The amount and the type of anesthetic agents were decided by the anesthesiologists and the surgeons. Continuous administration of phenylephrine was immediately started at 1 mg/hour to avoid hypotension due to anesthesia. Five milliliters of 0.25% ropivacaine was administered via the epidural catheter.

**Fig. 2:**
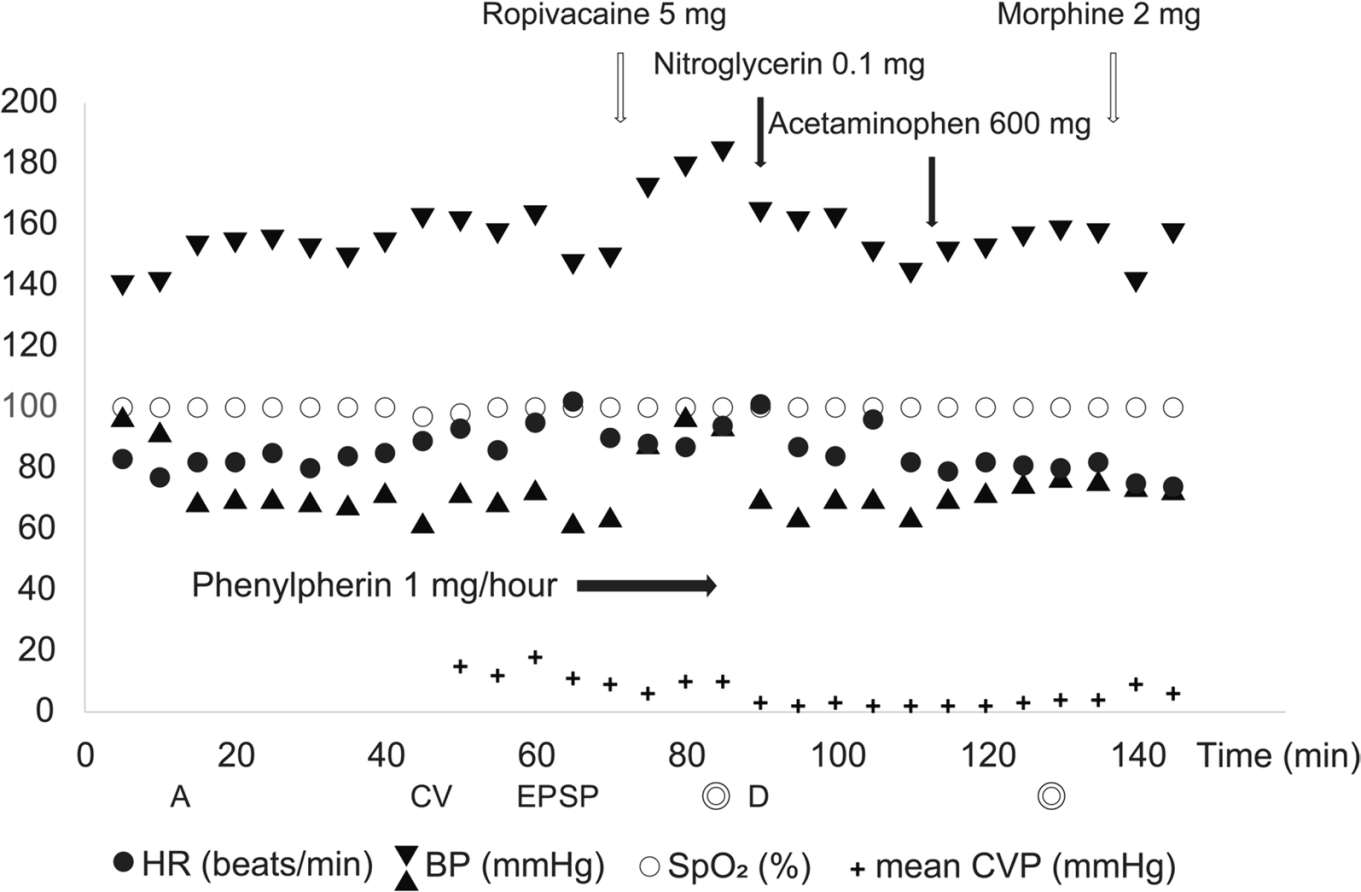
Anesthesia record from entry to departure from the operating room. Symbols and abbreviations: ◎, operation started/ended; **A**, arterial line inserted; CV, central venous line inserted; EP, epidural catheter inserted and test dose of 1% mepivacaine (3 ml) administered; SP, spinal anesthesia induced with 5 mg of 0.5% hyperbaric bupivacaine and 10 μg of fentanyl; D, delivery; HR, heart rate; BP, blood pressure; SpO_2_, saturation of percutaneous oxygen; CVP, central venous pressure. Black arrows: drug administered intravenously. White arrows: drug administered epidurally

After confirming sensory block at the Th4 level, the operation was started. Before delivery, 0.1 mg of nitroglycerin was administered to relax the uterus. The neonate was delivered 4 minutes after starting the cesarean section. Central venous and peripheral blood pressures were maintained during cesarean delivery. The patient did not report any nausea, pain, or dyspnea intraoperatively. After the delivery, 600 mg of acetaminophen was administered. Once the operation was finished without complications, 2 mg of morphine was administered via the epidural catheter, which was then removed. Operation and anesthesia times were 49 minutes and 88 minutes, respectively. Total infusion and blood loss volumes were 1050 ml and 1029 ml, respectively (Figs. 1, 2). Postoperatively, the patient was transferred to the ICU and hemodialysis was performed. The anesthetic record is shown in Fig. 2. She was transferred to the gynecology ward on postoperative day (POD) 5 and discharged on POD 10. Cardiac function improved after the cesarean section. As of the time of writing, both mother and child were doing well.

## Discussion

End-stage kidney disease (ESKD) is associated with low fertility, with rates of conception among women on dialysis estimated at 1% that of the general population [1]. On the other hand, deliveries from patients with ESKD have increased in recent years, partly due to improved treatment results [1, 2]. Because preterm delivery and cesarean section are reportedly more common in pregnancies complicated by ESKD, anesthesiologists are likely to be involved via deliveries such as cesarean sections, as in this case [2].

The major problem in the present case was the appearance of heart failure in a pregnant patient with existing chronic renal failure. To the best of our knowledge, there have not been any reports of cesarean sections for pregnancies complicated by renal failure with heart and respiratory failure. The management of pregnant women with either renal or heart failure is generally complex. Unsurprisingly then, formulating perinatal and anesthetic management in this case was difficult. Heart failure concomitant with renal failure is common [3]. Before she got pregnant, she had no episode of heart failure or disease. Although the reason heart failure developed in the present case remains unclear, changes in the circulating blood volume associated with pregnancy may have been involved. Fortunately, heart failure improved slightly and pulmonary edema decreased with intensive care that included strict fluid management. As a result, the patient could be managed until 24 weeks of gestation, when neonatal viability was expected to be sufficient to allow delivery [4]. In pregnant women with renal failure, the possibility of heart failure developing early in pregnancy might need to be considered, including in terms of the management of maintenance dialysis.

Cesarean section under general anesthesia has been reported to show a higher mortality rate for the patient than cesarean section under regional anesthesia [5]. Cesarean section is thus generally performed under regional, not general, anesthesia [6]. In pregnancies complicated by heart failure, anesthesia management with combined spinal and epidural anesthesia (CSEA) allows better pain management and control of circulatory fluctuations [7, 8]. CSEA was thus the first-choice anesthetic method in the present case. However, the method of CSEA should be considered with care in pregnant women on dialysis. Because an anticoagulant is needed to perform dialysis, the coagulation status of the patient had to be considered before inducing CSEA.

Nafamostat was used as the anticoagulant for dialysis in this patient. Nafamostat is a shorter-acting anticoagulant than heparin and is used for hemodialysis in perioperative patients [9, 10]. Dialysis with nafamostat may be better performed in patients requiring spinal or epidural anesthesia administration. Coagulability in the present patient was normal, so CSEA was feasible. In addition, anesthesia planning should consider the postoperative dialysis schedule. In the present patient, the epidural catheter was removed immediately after completion of the cesarean section. The patient therefore had sufficient time between removal of the epidural catheter and subsequent hemodialysis.

The patient was monitored by measuring the central venous pressure and arterial pressure. No significant changes in these pressures were seen after delivery. Women at risk of heart failure show a risk of decompensation after delivery, since aortocaval decompression and uterine involution can markedly increase preload [8]. In the present case, circulation was maintained after delivery because the cesarean section was performed after treatment for heart failure. Furthermore, the uterus was relatively small because the cesarean section was performed at 24 weeks of gestation, so little increase in blood flow was seen after delivery. Insertion of a Swan–Ganz catheter was considered but was not performed because the patient remained conscious under CSEA. We were concerned that the insertion of the Swan–Ganz catheter would cause pain and discomfort and consequently disrupt her hemodynamics. We were also concerned about the risks associated with use of the Swan–Ganz catheter such as increasing mortality [11].

On the other hand, CSEA might worsen hemodynamics compared with general anesthesia, so we were prepared to insert a Swan–Ganz catheter if needed. Monitoring with a Swan–Ganz catheter might have been required if her hemodynamics worsened. Whether or not to insert a Swan–Ganz catheter should be considered in each patient.

The expected complications of cesarean section were hypotension and nausea. The overall incidence of nausea and vomiting during regional anesthesia for cesarean delivery is high [12]. Hypotension is one cause of nausea, and nausea and vomiting might in turn alter the hemodynamics [13]. In recent years, administration of phenylephrine has been promoted for cesarean sections performed under spinal anesthesia to avoid hypotension [12]. In the present case, infusion of phenylephrine stabilized blood pressure and the cesarean section was finished without any need for administration of antiemetics. On the other hand, administrating antiemetics prophylactically in the cesarean section under local anesthesia is recommended recently [12]. Antiemetics like ondansetron could have been administered prophylactically in this case.

In the present case, the epidural catheter had to be removed at the end of surgery because dialysis was scheduled for immediately after surgery. Morphine was administered before catheter removal to provide postoperative analgesia. In general, morphine administration is contraindicated in patients with renal failure due to the risk of respiratory depression [14]. However, the possibility of inadequate analgesia exacerbating cardiac and respiratory insufficiency was a concern in this case. On the other hand, the patient was scheduled to enter the ICU postoperatively and respiratory status was being closely monitored. Although morphine was administered via the epidural catheter, the use of morphine in similar cases requires careful consideration not only by anesthesiologists but also by obstetricians and gynecologists providing postoperative management.

## Conclusion

Cesarean section in this pregnant woman with renal and heart failure was safely performed using CSEA. When a cesarean section is performed on a pregnant woman with complications such as renal or cardiac failure, a detailed anesthesia plan should be elaborately developed in consultation with obstetricians, cardiologists, urologists, and anesthesiologists.

## Data Availability

The datasets used during the current case are available from the corresponding author upon reasonable request.

## References

[1] Oliverio AL, Hladunewich MA. End-stage kidney disease and dialysis in pregnancy. Adv Chronic Kidney Dis. 2020;27:477–85. 33328064 10.1053/j.ackd.2020.06.001 PMC7781109

[2] Oliverio AL, Bragg-Gresham JL, Admon LK, *et al*. Obstetric deliveries in US women with ESKD: 2002–2015. Am J Kidney Dis. 2020;75:762–71. 31785826 10.1053/j.ajkd.2019.08.029 PMC7183877

[3] Joseph MS, Palardy M, Bhave NM. Management of heart failure in patients with end-stage kidney disease on maintenance dialysis: a practical guide. Rev Cardiovasc Med. 2020;21:31–9. 32259902 10.31083/j.rcm.2020.01.24

[4] Ancel PY, Goffinet F, Kuhn P, *et al*. Survival and morbidity of pretearm children born at 22 through 34 weeks’ gestation in France in 2011: results of the EPIPAGE-2 cohort study. JAMA Pediatr. 2015;169:230–8. 25621457 10.1001/jamapediatrics.2014.3351

[5] Hawkins JL, Chang J, Palmer SK, Gibbs CP, Callaghan WM. Anesthesia-related maternal mortality in the United States: 1979–2002. Obstet Gynecol. 2011;117:69–74. 21173646 10.1097/AOG.0b013e31820093a9

[6] Practice Guidelines for Obstetric Anesthesia. An updated report by the American society of anesthesiologists Task Force on Obstetric Anesthesia and the Society for Obstetric Anesthesia and Perinatology. Anesthesiology. 2016;124:270–300. 26580836 10.1097/ALN.0000000000000935

[7] Ogata J, Horishita T, Shiraishi M, Minami K. Combined spinal-epidural anesthesia for cesarean section in a patient with Takayasu arteritis complicated by heart failure. J Anesth. 2007;21:525–6. 18008128 10.1007/s00540-007-0547-5

[8] Meng ML, Arendt KW. Obstetric Anesthesia and Heart Disease: practical clinical considerations. Anesthesiology. 2021;135:164–83. 34046669 10.1097/ALN.0000000000003833 PMC8613767

[9] Nakamura Y, Tada K, Matsuta M, Murai A, Tsuchiya H. Anaphylactic reactions caused by nafamostat mesylate during hemodialysis before surgery for carpal tunnel syndrome. Case Rep Nephrol. 2021;2021:1148156. 35003816 10.1155/2021/1148156 PMC8731279

[10] Akizawa T, Koshikawa S, Ota K, *et al*. Nafamostat mesilate: a regional anticoagulant for hemodialysis in patients at high risk for bleeding. Nephron. 1993;64:376–81. 8341382 10.1159/000187357

[11] Connors AF Jr, Speroff T, Dawson NV, *et al*. The effectiveness of right heart catheterization in the initial care of critically ill patients. SUPPORT Investigators. JAMA. 1996;276:889–97. 8782638 10.1001/jama.1996.03540110043030

[12] Macones GA, Caughey AB, Wood SL, *et al*. Guidelines for postoperative care in cesarean delivery: Enhanced Recovery After Surgery (ERAS) Society recommendations (part 3). Am J Obstet Gynecol. 2019;221(247):e1–9. 10.1016/j.ajog.2019.04.012 30995461

[13] Ituk U, Habib AS. Enhanced recovery after cesarean delivery. F1000Res. 2018;7:F1000. 29770203 10.12688/f1000research.13895.1 PMC5931266

[14] Dean M. Opioids in renal failure and dialysis patients. J Pain Symptom Manage. 2004;28:497–504. 15504625 10.1016/j.jpainsymman.2004.02.021

